# A simple fluorescence based assay for quantification of human immunodeficiency virus particle release

**DOI:** 10.1186/1472-6750-10-32

**Published:** 2010-04-20

**Authors:** Johannes Hermle, Maria Anders, Anke-Mareil Heuser, Barbara Müller

**Affiliations:** 1 Department of Infectious Diseases, Virology, University Hospital Heidelberg, Im Neuenheimer Feld 324, D-69120 Heidelberg, Germany

## Abstract

**Background:**

The assembly and release of human immunodeficiency virus (HIV) particles from infected cells represent attractive, but not yet exploited targets for antiretroviral therapy. The availability of simple methods to measure the efficiency of these replication steps in tissue culture would facilitate the identification of host factors essential for these processes as well as the screening for lead compounds acting as specific inhibitors of particle formation. We describe here the development of a rapid cell based assay for quantification of human immunodeficiency virus type 1 (HIV-1) particle assembly and/or release.

**Results:**

Using a fluorescently labelled HIV-derivative, which carries an eYFP domain within the main viral structural protein Gag in the complete viral protein context, the release of virus like particles could be monitored by directly measuring the fluorescence intensity of the tissue culture supernatant. Intracellular Gag was quantitated in parallel by direct fluorescence analysis of cell lysates, allowing us to normalize for Gag expression efficiency. The assay was validated by comparison with p24 capsid ELISA measurements, a standard method for quantifying HIV-1 particles. Optimization of conditions allowed the robust detection of particle amounts corresponding to 50 ng p24/ml in medium by fluorescence spectroscopy. Further adaptation to a multi-well format rendered the assay suitable for medium or high throughput screening of siRNA libraries to identify host cell factors involved in late stages of HIV replication, as well as for random screening approaches to search for potential inhibitors of HIV-1 assembly or release.

**Conclusions:**

The fast and simple fluorescence based quantification of HIV particle release yielded reproducible results which were comparable to the well established ELISA measurements, while in addition allowing the parallel determination of intracellular Gag expression. The protocols described here can be used for screening of siRNA libraries or chemical compounds, respectively, for inhibition of HIV in a 96-well format.

## Background

**Figure 1:**
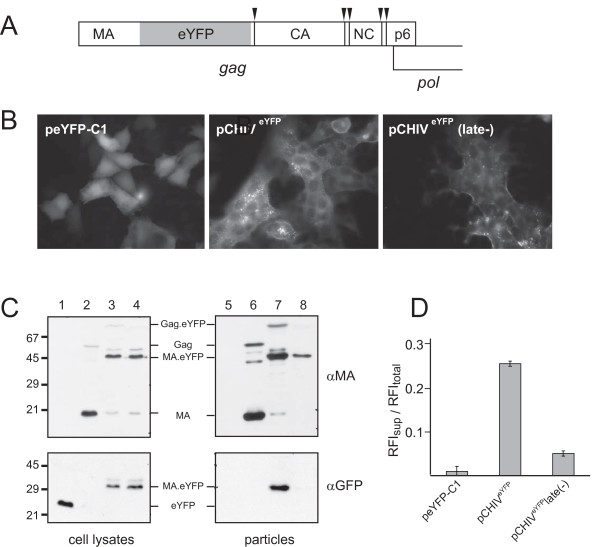
**Characterization of the fluorescently labelled virus derivatives**. **(A) **Map showing the insertion of the *eyfp *gene within the *gag *open reading frame of pCHIV. Arrowheads indicate cleavage sites for HIV protease. **(B) **Fluorescence micrographs of HeLaP4 cells transfected with plasmids peYFP-C1, pCHIV^eYFP ^or pCHIV^eYFP^late(-), respectively, recorded at 40 h post transfection. Note that a shorter exposure time was used for cells transfected with peYFP-C1 to adjust for the higher cytoplasmatic fluorescence intensity. **(C) **Immunoblot analysis of cell lysates and VLPs purified from the supernatant of transfected cells. At 36 h post transfection with peYFP-C1 (lanes 1 and 5), pCHIV (lanes 2 and 6), pCHIV^eYFP ^(lanes 3 and 7) or pCHIV^eYFP^late(-) (lanes 4 and 8), respectively, cells were harvested and particles were prepared from the medium by ultracentrifugation through a sucrose cushion. Samples corresponding to equal cell numbers or equal amounts of supernatant were subjected to SDS-PAGE, transferred to a nitrocellulose membrane and proteins were detected by enhanced chemiluminescence using the indicated antisera. Antiserum raised against GFP displayed cross-reactivity against eYFP. **(D) **Relative release efficiencies of eYFP, HIV^eYFP ^and HIV^eYFP^late(-) particles. Relative eYFP fluorescence intensities of tissue culture supernatants and lysates of cells transfected with the indicated plasmids were determined at 44 h post transfection using a TECAN Safire multi-well reader. Values shown here were obtained by subtracting the background derived from untransfected cells and dividing extracellular by total (intracellular plus extracellular) fluorescence. Data shown represent mean values and standard deviations from three parallel transfections.

The acquired immunodeficiency syndrome (AIDS), caused by infection with the human immunodeficiency virus (HIV), is a major cause of disease and death worldwide. In the absence of a protective vaccine, prevention of infection and the treatment with antiretroviral drugs remain the only options to prevent the spread of HIV and combat the disease. Current therapy of HIV infection (highly active antiretroviral therapy, HAART) involves treatment with a combination of three or more drugs targeting different steps in HIV replication [reviewed in [1]]. The benefits of HAART are limited by viral resistance development and the transmission and spread of resistant HIV variants. Therefore, additional treatment options targeting alternative steps in the viral replication pathway are needed. The late replication steps of virion assembly and release are not targeted by any of the currently approved antiretroviral drugs and thus present targets for alternative inhibition strategies. The processes of HIV particle assembly and release, as well as the morphological maturation of particles into infectious virions, are orchestrated by the main structural polyprotein Gag. Compounds that do interfere with HIV infectivity in tissue culture by interaction with the Gag polyprotein or its capsid domain (CA), respectively, have been described. The compounds bevirimat [[2]; reviewed in [3]] and CAP-1 [4,5], do not impair HIV immature particle release, but affect the maturation into infectious particles by binding to a proteolytic processing site in the Gag polyprotein precursor or to the N-terminal domain of the CA protein, respectively. Furthermore, a peptide (CAI), which acts as a *bona fide *inhibitor of particle assembly from purified HIV structural protein *in vitro *has been reported [6,7]. A cell-permeable derivative of this peptide has been shown in a proof of principle study to inhibit HIV replication in tissue culture, albeit with IC50 values in the low to mid μM range and a low selectivity index [8]. Small molecule chemical compounds which specifically inhibit the steps of immature Gag assembly or particle release have not yet been identified. Rational development of such compounds is hindered by the fact that the late stages in the viral life cycle are highly complex processes involving the interaction of viral components with intricate cellular machineries, which are currently only partly understood. It is well established that components of the cellular ESCRT machinery [9], which are recruited by a so-called 'late domain' motif within Gag, are involved in the budding of HIV particles from the host cell [for review see [10]] and a number of additional cellular factors have been implicated to be involved in the late stages of HIV replication [reviewed in [11,12]]. However, a comprehensive picture of cellular factors and pathways involved in the transport, assembly and release of viral components is currently lacking. Genome-wide siRNA screening approaches have been carried out with the aim of identifying host cell factors essential or inhibitory for HIV replication [13-15]. So far, only two published studies include late stages of virus replication in their analysis, and the results from the different studies showing surprisingly little overlap [16-18]. The availability of simplified cell based assay procedures specifically monitoring the steps of HIV assembly and particle release in tissue culture would greatly facilitate both large-scale siRNA analyses focussing in particular on the late stages of virus replication, as well as the random screening of chemical compound libraries in order to identify lead substances which interfere with HIV particle formation. A well established and widely used method for quantification of HIV particle production is an enzyme linked immunosorbent assay (ELISA) detecting the viral capsid (CA) protein p24 in tissue culture supernatants. However, ELISA measurements display a limited linear range and require many handling steps and expensive reagents. In contrast, measurement of fluorescence intensity is a direct, quantitative readout which can easily be performed in a multi-well format. Thus, we decided to establish a simplified assay based on our previously described fluorescently labelled HIV derivative carrying an eGFP moiety inserted as a separate domain into the main viral structural polyprotein Gag [[19,20]; Figure 1A]. Cells transfected with the *egfp*-carrying proviral construct release fluorescent virus like particles in a late domain dependent manner and we have previously used these labelled derivatives to analyze the dynamics of HIV-1 particle formation [21]. Here, we describe a simple and direct assay for monitoring HIV particle release through quantitation of fluorescent VLPs in tissue culture supernatant. Based on this principle, we have established protocols suitable for the screening of either siRNAs or chemical compounds for their interference with HIV assembly and/or release in a multi-well, medium to high throughput format.

## Results and discussion

### Setup of the assay procedure and optimization of assay conditions

The aim of this study was to develop a simple and direct assay for HIV-1 release based on our previously characterized fluorescently labelled HIV derivative [19,20], which should allow the quantitation of particles in tissue culture supernatants from virus producing cells by direct measurement of fluorescence intensity. Towards this end, we first optimized the detection conditions to increase the sensitivity of the measurements in tissue culture supernatants. Due to intrinsic fluorescence of tissue culture media components at the wavelengths used for detection of fluorescent proteins, optimization of the assay initially required the selection of the fluorophore best suited for detection against medium background. Fluorescence measurements of serial dilutions of bacterially expressed, purified enhanced green, cyan and yellow fluorescent proteins (eGFP, eYFP, eCFP) as well as monomeric red fluorescent protein [22] in PBS and various tissue culture media indicated that enhanced yellow fluorescent protein eYFP was most suitable among these fluorophors for detection against complete medium background. EYFP concentrations above10 ng/ml were detectable over medium background (Additional file 1, Figure S1). Omission of phenol red or fetal calf serum from the medium did not significantly alter the sensitivity of detection.

Based on these findings, we constructed an HIV derivative carrying the *eyfp *gene within the *gag *open reading frame, analogous to the previously described construct pCHIV^eGFP ^[20]. The fluorescent protein was inserted between the matrix (MA) and capsid (CA) domains of the Gag polyprotein, upstream of the proteolytic processing site between MA and CA (Figure 1A). Transfection of cells with pCHIV and derivatives results in expression of all viral proteins, except for Nef, under the control of the CMV promoter and to formation and release of entry competent virus like particles (VLPs). The genome encoded by pCHIV is replication incompetent due to deletion of the complete viral long terminal repeat regions essential for reverse transcription and integration.

As shown in Figure 1B, cells transfected with pCHIV^eYFP ^displayed the expected yellow fluorescence. Furthermore, HeLaP4 cells which express the receptor and co-receptors of HIV formed large syncytia upon transfection with pCHIV derived constructs, indicative of HIV Env protein expression. Cell lysates as well as particle preparations from the supernatant of transfected cells were prepared and analyzed by immunoblot (Figure 1C). Analogous to our results obtained with the related construct pKHIV^eGFP ^[19], the Gag.eYFP fusion protein expressed in the viral context was processed upon particle maturation by the viral protease to yield MA.eYFP, and released pelletable particles containing the MA.eYFP fusion protein in a late domain dependent manner (Figure 1C and data not shown). Accordingly, particles should be detectable in the tissue culture supernatant by measurement of eYFP fluorescence intensity.

### Specific detection of released particles by fluorescence measurements

The exploitation of fluorescent MA for the quantitation of specific particle release from cells transfected with pCHIV^eYFP ^by fluorescence measurements required appropriate controls. The fluorescence readout - analogous to p24 determination by ELISA - does not necessarily reflect the amount of complete particles released, but rather quantitates the amount of the main particle component Gag present in tissue culture supernatants. Consequently, unspecific release of free Gag, p24 CA or MA.eYFP from damaged or lysed cells could impair the accuracy of results obtained by either ELISA or fluorescence based measurements. To control for unspecific protein release due to cell damage, we used cells expressing unfused eYFP protein, which is not secreted from intact cells, but will be detected in the medium when the sample contains cell debris or cytoplasmic content from lysed cells. Secondly, a positive control for the successful inhibition of release was established. For this purpose, we generated a release deficient variant of the fluorescently labelled HIV derivative by replacing the 'late domain' motif PTAP within the p6 domain of Gag.eYFP with the amino acid sequence LIRL. The late domain is known to mediate interaction with the host cell ESCRT machinery [see [10] for review] and its mutation to LIRL has been previously shown to affect HIV release efficiency [23]. An inhibitory compound which directly blocks viral late domain - ESCRT interaction or an siRNA which downmodulates expression of a late domain interacting host factor can be expected to yield a comparable decrease in release efficiency as the mutation of the late domain, while compounds or siRNAs targeting the Gag assembly process may theoretically accomplish even stronger reduction.

Expression of the Gag.eYFP.late(-) fusion protein and the release deficiency of this construct was confirmed by immunoblot analysis of cell lysate and pelleted tissue culture supernatant (Figure 1C). While comparable amounts of Gag.eYFP and Gag.eYFP.late(-) were detected in cell lysates (compare lanes 3 and 4), the amount of Gag derived products in pelleted particles was significantly decreased in the case of the late-variant (compare lanes 7 and 8). Free eYFP was expressed in transfected cells (lane 1), but not detected in pelleted supernatant (lane 5). We then determined the relative release efficiency of Gag.eYFP.late(-) by fluorescence measurements.

Figure 1D shows the result of a comparison of release efficiencies between 293T cells transfected with plasmids pEYFP-C1, pCHIV and pCHIV^eYFP^late(-). At 48 h post transfection tissue culture supernatants were harvested and cells were lysed by detergent treatment as described in methods. Supernatants from particle producing cells displayed typical spectroscopic characteristics of eYFP with excitation and emission maxima at 512 nm and 527 nm (Additional file 2, Figure S2). Relative release efficiencies were calculated by dividing fluorescence intensities measured in tissue culture supernatants by the corresponding total fluorescence intensities measured in supernatants and cell lysates. Cells transfected with plasmid peFYP-C1 displayed high intracellular fluorescence but the relative amount of eYFP released into the supernatant was low in comparison to cells transfected with pCHIV^eYFP^. Cells expressing the late domain defective variant of the labelled virus reproducibly showed an approximately five-fold reduction of relative release efficiency as compared to cells expressing the wild-type variant.

### Sensitivity of measurements

Our previous characterization of eGFP labelled HIV derivatives had revealed that - while bulk release efficiency of labelled virus was comparable to wild-type - cells expressing the labelled HIV derivative displayed electron dense aggregates at the plasma membrane, suggesting altered release kinetics; furthermore released particles displayed reduced infectivity. Full wild-type particle infectivity and budding site morphology was restored by co-transfection of an equimolar amount of wild-type HIV proviral plasmid, leading to the formation of mixed particles carrying 50% of labelled Gag [19]; assuming an average number of approximately 2400 molecules of Gag per particle [24], particles contain thus approximately 1200 molecules of fluorescent protein. For this reason, equimolar mixtures of eYFP labelled proviral derivatives with the corresponding non-labelled provirus were used in all further experiments.

**Figure 2:**
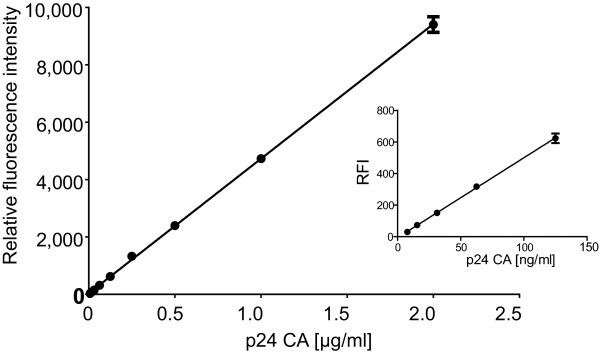
**Titration of eYFP labelled VLPs**. Mixed particles were purified from the tissue culture supernatant of 293T cells transfected with an equimolar mixture of pCHIV and pCHIV^eYFP ^by centrifugation through a sucrose cushion and their concentration determined by p24 ELISA. The relative eYFP fluorescence intensity of serial dilutions in DMEM/10% FCS was determined using a TECAN Saphire multi-well reader. Background fluorescence from the medium was subtracted and fluorescence intensities of the samples were plotted as a function of p24 CA concentration. The plot shows mean values and standard deviation from triplicate dilutions; the line represents a linear regression. The inset shows an expansion of the lower part of the same data set. The line represents a linear regression to the data points shown.

In order to determine the sensitivity of fluorescence based particle quantification, VLPs were prepared from the tissue culture supernatant of cells co-transfected with an equimolar mixture of pCHIV and pCHIV^eYFP ^by ultracentrifugation through a sucrose cushion. The amount of CA p24 present in the particle preparation was determined by ELISA, which represents a currently used standard method for HIV particle quantification. Since both eYFP and p24 CA are expressed from pCHIV^eYFP ^as parts of the Gag polyprotein, they are translated and packaged into VLPs at equimolar amounts; in the case of the mixed particles used here, one molecule of eYFP will thus correspond to two molecules of CA. Serial dilutions of fluorescent particles in complete medium were set up and their relative eYFP fluorescence intensity was determined (Figure 2). The lowest particle concentration detectable above background by fluorescence measurements in complete medium corresponded to approximately 15-30 ng CA/ml (equivalent to ~8-15 ng eYFP/ml), which was in good agreement with the results from measurements using purified fluorescent proteins. Omission of FCS or phenol red from the medium or replacement of the medium by PBS only resulted in a minor increase in sensitivity (data not shown). From these and similar data sets we concluded that robust fluorescence intensity quantitation can be performed for particle concentrations corresponding to 50 ng p24/ml or higher. This sensitivity is significantly lower than that of advanced ELISA based detection systems [25]. However, this does not affect the usefulness of the system for the intended applications, since the amounts of particles released under optimized assay conditions were in a range easily detectable by fluorescence measurements (see below).

### Assay validation and time course of particle release

**Figure 3:**
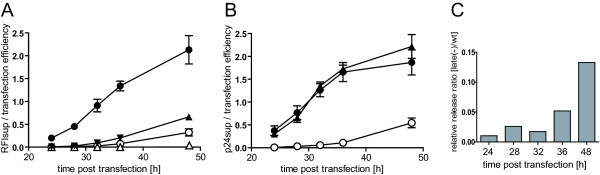
**Time course of labelled VLP release cells determined by fluorescence measurement or ELISA, respectively**. 293T cells were transfected with mixtures of pCHIV/pCHIV^eYFP ^(filled circles) or pCHIVlate(-)/pCHIV^eYFP^late(-) (open circles), respectively. Cells transfected with peYFP-C1/empty vector (open triangles) or peYFP/pCHIV (filled triangles) served as controls. At the indicated time points after transfection, tissue culture supernatants were sampled and analyzed in parallel by fluorescence measurements **(A) **or ELISA **(B)**, respectively, as described in methods. To control for transfection efficiency, cell lysates prepared at the end of the experiment were subjected to the same analyses. Normalized release efficiency was determined by subtracting the background derived from untransfected cells and dividing extracellular fluorescence or p24 CA versus intracellular fluorescence or p24 CA determined at 48 h post transfection, respectively. Data represent mean values and standard deviations from triplicate transfections. **(C) **Increase of unspecific release upon prolonged incubation of cells. Normalized release values for HIVlate(-)/HIV^eYFP^late(-) determined in the experiment shown in Figure 3A at the indicated times post transfection were divided by the respective values obtained for HIV/HIV^eYFP^.

We then analysed the time course of fluorescence release from cells transfected with equimolar mixtures of either wild-type or late domain defective variants of pCHIV/pCHIV^eYFP^, respectively. Samples of tissue culture supernatant were removed at consecutive time points between 24 and 48 h post transfection and subjected to fluorescence measurements. Cells transfected with peYFP-C1 were used as a negative control. In order to investigate whether budding of HIV from the membrane could promote the release of free eYFP from cells, we included cells transfected with a mixture of pCHIV and peFYP-C1 as an additional control. At 48 h post transfection, cells were lysed by detergent treatment and the fluorescence intensity of the lysate determined in order to normalize for transfection efficiency and Gag or eYFP expression in the different samples. As shown in Figure 3A, fluorescence in the supernatant above background was detectable at 24 h post transfection with pCHIV/pCHIV^eYFP ^(filled circles) and increased over the following hours. Again, release from the late(-) variant (open circles) was found to be diminished. While fluorescence in the supernatant of peYFP-C1 transfected cells (open triangles) remained at background levels, release of low levels of free eYFP was promoted in the presence of HIV expression (filled triangles). This might be explained by unspecific packaging of cytosolic eYFP into budding virus particles, since we observed that in this case release was also influenced by the presence of a functional HIV late domain (data not shown).

Results obtained from fluorescence measurements were validated by determination of p24 CA in the same samples by ELISA. Again, we calculated the amount of p24 in the supernatant relative to the amount measured in cell lysates at 48 h. Results are shown in Figure 3B. Similar concentrations of p24 were detected in supernatants from cells transfected with pCHIV/pCHIV^eYFP ^(filled circles) and with pCHIV/peYFP-C1 (filled triangles), demonstrating that insertion of eYFP within Gag did not affect release efficiency; p24 concentrations measured corresponded to ~70 ng/ml at 24 h post transfection and ~0.5 μg/ml at the end of the experiment, respectively. Evaluation by ELISA yielded similar results for pCHIV/pCHIV^eYFP ^(filled circles) and pCHIVlate(-)/pCHIV^eYFP^late(-) (open circles) as the fluorescence based assay (compare Figure 3A and Figure 3B), confirming the validity of the fluorescence assay method. A slightly larger deviation between parallel samples was observed in the ELISA experiment, presumably because this procedure involved more handling steps. The observation that the relative ratio of intra- to extracellular fluorescence differs somewhat between the two types of measurement is explained by the fact that unprocessed Gag (which represents the predominant form in cell lysates) is recognized less well than processed CA (the predominant form in particles), by immunoprecipitation [26] or ELISA. Even prior heat and detergent treatment of samples, which has been reported to make unprocessed Gag accessible to ELISA detection [27], did not confer full reactivity of the precursor form in our hands. Thus, data derived from the ELISA measurements are less suitable for reliable normalization of CA levels relative to intracellular Gag expression levels. In contrast, in vitro cleavage of immature fluorescent particles using purified PR did not result in a change in fluorescence intensity, demonstrating that the fluorescence based quantitation of Gag was independent of its processing status (data not shown). We concluded that the assay described here is suitable to monitor HIV-1 particle release and can be used to test inhibition of HIV-1 particle formation in tissue culture.

In order to define conditions yielding efficient late domain dependent release accompanied by low levels of unspecific release we compared the ratio between release of the wild-type and late(-) virus variants at the different time points. The ratio of late(-) particles released compared to wild-type particles increased at later time points, presumably due to unspecific Gag release from damaged or dead cells upon prolonged protein over-expression (Figure 3C and data not shown), indicating that harvest times of 33-36 h post transfection were optimal under the conditions used.

### Adaptation of the assay to medium throughput formats

**Figure 4:**
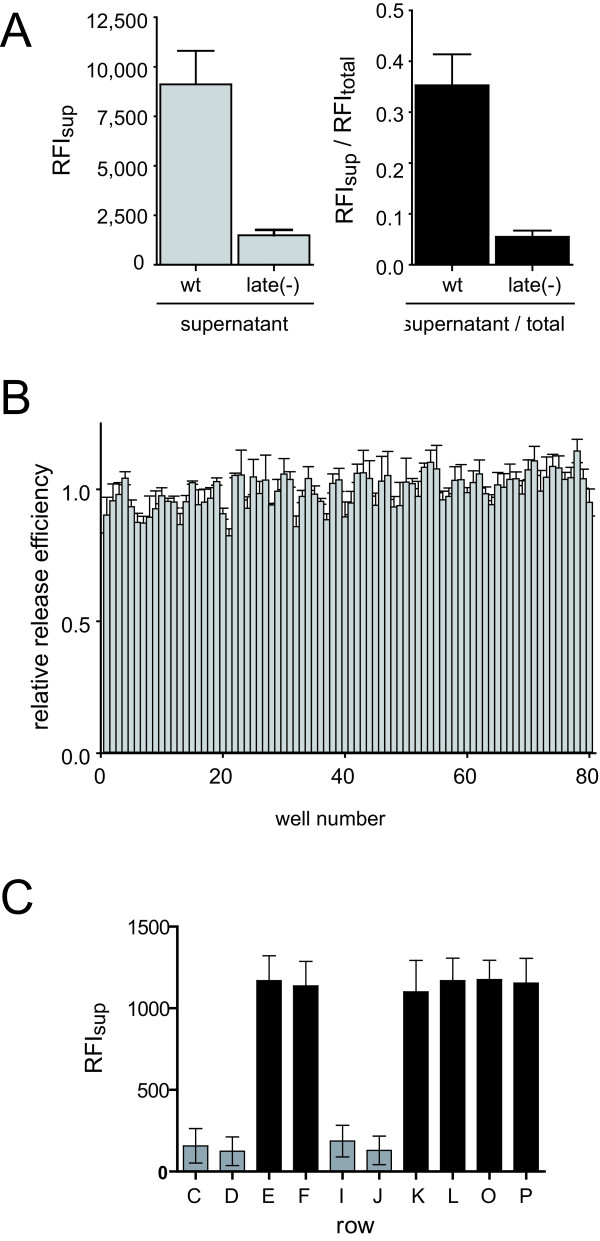
**Adaptation of the assay to a multi-well format**. **(A) **Reproducibility of controls. 293T cells transfected with an equimolar mixture of pCHIV and pCHIV^eYFP ^(wild-type) or their late domain defective variants (late(-)) were seeded into 96-well plate. Fluorescence intensities of tissue culture supernatants (grey bars) or release efficiencies (supernatant fluorescence intensities divided by total fluorescence intensity (supernatant and cell lysate; black bars) were determined as described in methods. Bars represent mean value and standard deviation of 176 wells from 88 plates. **(B) **Variability across a 96-well plate. 293T cells transfected with an equimolar mixture of pCHIV and pCHIV^eYFP ^were seeded into 96-well plate. 0.5% of DMSO was added to the medium at 14 h post transfection to mimic the addition of compounds dissolved in DMSO. Supernatants and cell lysates were harvested at 36 h post transfection and relative release efficiencies were determined by dividing extracellular by total fluorescence and normalized by dividing through the median of release efficiencies from all wells of the respective plate. Mean values and standard deviations from three 96-well plates are shown. **(C) **Variability across a 384-well plate. 293T cells transfected with a mixture of pCHIV/pCHIV^eYFP ^(black bars) or their late domain defective variants (grey bars), respectively, were seeded into a 384-well plate. Relative fluorescence intensities of the supernatants were measured at 34 h post transfection. Mean values and standard deviations from 24 wells each are shown.

Further adaptation of the assay to a semi-automated multi-well format was performed to render the assay suitable for random screening. Two different protocols were established suitable for screening of chemical compound libraries or for siRNA screening approaches, respectively. For the purpose of compound screening we established a protocol for the bulk transfection of 293T cells. Briefly, 293T cells were harvested and transfected in suspension using equimolar mixtures of pCHIV/pCHIV^eYFP^, pCHIVlate(-)/pCHIV^eYFP^late(-), or plasmid peYFP-C1 alone, respectively. Cell suspensions were then distributed into wells of a 96-well plate or 384-well plate, respectively, using an automated reagent dispenser and plates were incubated at 37°C. The addition of chemical compounds dissolved in DMSO was mimicked by the addition of a final concentration of 0.5% DMSO to the medium at 14 h post transfection. At 36 h post transfection, plates were briefly spun to pellet potential cell debris. Aliquots of tissue culture supernatants were transferred to fresh plates using an automated liquid handler, the remaining supernatant was removed from the cell layer and cells were lysed on the plate as described in methods. Relative fluorescence intensities of supernatants and cell lysates were determined using a TECAN Safire multi-well reader. Supernatants and lysates from untransfected cells were used to determine background fluorescence. As shown in Figure 4 and Additional file 3, Figure S3, this procedure yielded robust results in 96-well plates. A limited data set of 60 positive (cells transfected with pCHIV/pCHIV^eYFP^, 0.5% DMSO) and 36 negative (untransfected cells) control wells from a pilot screen including 6 replicate plates was used to estimate statistical effect size, yielding a Z factor [28] of 0.71 (Additional file 3, Figure S3). Mean fluorescence intensities in the supernatant of 176 wells from 88 plates (Figure 4A, grey bars) showed low plate-to-plate variability. The amount of eYFP labelled protein released into the supernatant by the late domain defective variant was reduced to 16% of the wild-type control. Very similar results were obtained when fluorescence intensities of the supernatant normalized to the total fluorescence measured in the supernatant and cell lysate were compared (Figure 4A, black bars). Thus, normalization for intracellular protein expression was not necessary to improve the accuracy of measurements in a standardized 96-well setup. In the context of a random screening assay, however, the parallel determination of intracellular eYFP intensity in a screening assay can serve to identify compounds which do not specifically interfere with HIV particle formation but rather exert a general effect on protein expression. Comparison of relative release efficiencies determined for labelled wild-type particles across rows 2-11 of 96-well plates in the absence of inhibitory compounds did not reveal any systematic aberration across the plate (Figure 4B). We conclude that the assay allows for medium throughput screening of potential inhibitors of HIV-1 assembly or release.

Transfer of the protocol to a 384-well format yielded less robust results (Figure 4C). Due to difficulties in reproducible preparation and measurement of cell lysates in the small well format, normalization of supernatant fluorescence to total fluorescence led to larger variability between wells (data not shown). While the assay in its present form could be used to determine relative amounts of Gag released into the supernatant using a 384-well format, further optimization would be required for a reliable parallel quantification of intracellular Gag.

**Figure 5:**
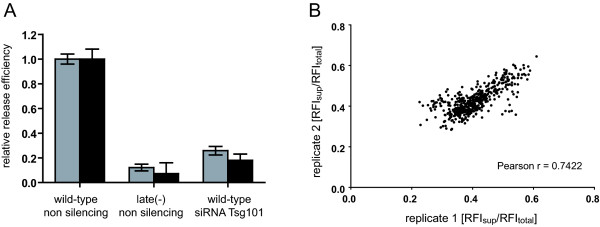
**Adaptation of the assay for an siRNA screening approach**. **(A) **Reduction of relative release efficiency by knock-down of Tsg101. 96-well plates were coated with non-silencing control siRNA or siRNA targeting Tsg101, respectively, as described in methods. 293T cells were seeded onto those plates for reverse transfection and incubated for 18 h at 37°C. Subsequently, cells were transfected with a mixture of pCHIV/pCHIV^eYFP ^or their late domain defective variants, respectively. Supernatants and cell lysates were harvested at 36 hours after this second transfection and relative fluorescence intensities were determined. Relative release efficiencies (RFI_sup_/RFI_total_) were normalized to the mean of the non-silencing control. Mean values and standard deviations from triplicate transfections are shown; black bars and gray bars, respectively, represent results from two independent experiments. **(B) **Reproducibility of results obtained in a pilot siRNA screen. The graph shows the correlation between normalized eYFP intensities (eYFP intensity of supernatant divided by total eYFP intensity; RFI_sup_/RFI_total_) of two replicates of an RNAi screen investigating host cell functions in HIV-1 assembly and release, comprising a total of 480 wells in 96-well plates. Each dot represents an individual siRNA mediated gene knockdown. The Pearson's correlation coefficient was calculated using GraphPad Prism.

Finally, a modified protocol was established to be used in screening of siRNA libraries for factors involved in HIV release. For this, 293T cells were distributed onto 96-well plates which had been pre-coated with an siRNA containing transfection mix using a previously described reverse transfection protocol [29,30]. Dried pre-coated plates could be either used directly for knockdown experiments or be stored for up to 15 months without any loss of efficacy, thus ensuring improved reproducibility and comparability between different plates of the same batch as compared to liquid transfection of siRNA. 293T cells were seeded onto these plates and incubated in order to achieve target gene knockdown, followed by transfection with pCHIV/pCHIV^eYFP ^and further incubation. In order to validate this approach we performed siRNA mediated knockdown of the cellular ESCRT protein Tsg101, which has been previously shown to impair HIV release [31]. At the conditions used, prolonged knock-down of Tsg101 resulted in reduced cell viability and cell death; to avoid this, we chose a relatively short incubation period of 18 h for the initial transfection with siRNA, followed by transfection with pCHIV/pCHIV^eYFP^. Incubation was then continued for 36 h before read out of fluorescence intensities in supernatant and cell lysates was carried out. As shown in Figure 5A, siRNA mediated knock-down of Tsg101 resulted in an approximately 4-5 fold reduction in release efficiency under these conditions, which was only slightly less pronounced than the reduction observed using the late(-) virus variant.

We have performed a pilot screen, in which we tested an siRNA library (*silencer *siRNAs, Ambion, Applied Biosystems) targeting approximately 740 human kinases with one individual siRNA per well and three distinct siRNAs per target gene. Since individual siRNAs from this library have not been tested for their silencing properties and time course of gene silencing, we chose longer incubation times of 30 h after the initial transfection with siRNA and 42 h after the second transfection. The results of this screen and the validation of primary hits will be described elsewhere. Exemplary for the reproducibility of results obtained using the established protocol, Figure 5B shows the correlation of relative release values (RFI_sup_/RFI_total_) obtained for two replicate sets of 480 wells from the siRNA screen. The values obtained for RFI_sup_/RFI_total _were slightly higher than those observed for the compound screen setup due to the modified assay procedure. Pearson's correlation coefficients between replicates of identical siRNA sets were generally found to be in the range of 0.7 - 0.8, indicating that the setup described here allowed us to obtain reproducible results in a 96 well format.

## Conclusion

We conclude that direct measurement of fluorescence intensities after transfection of cells with a labelled HIV derivative represents a reliable method for monitoring HIV particle release, which is suitable for use in random screening approaches to search for chemical inhibitors or siRNAs specifically interfering with HIV assembly or release.

## Methods

### Plasmids

Plasmid pCHIV^eYFP ^was constructed by inserting a PCR fragment comprising the complete eYFP coding sequence from peYFP-C1 (Invitrogen) into an engineered ClaI-site between the codons for amino acids 128 and 129 of MA. The cloning strategy for this construct, as well as for its late domain defective variant was analogous the cloning strategy previously described for eGFP labelled virus variants [19,20]. Plasmids for the expression of recombinant His-tagged eGFP, eYFP and eCFP were generated by cloning PCR fragments comprising the respective coding sequences into the NdeI and BamHI sites of pET16b (Novagen). Plasmid pmRSETmRFP1 was a kind gift of R. Tsien.

### Tissue culture and virus preparation

293T and HeLaP4 cells were cultured at 37°C, 5% CO2 in Dulbecco's modified Eagle's medium (DMEM) supplemented with 10% fetal calf serum (FCS), penicillin, streptomycin, 4 mM glutamine and 10 mM Hepes. For viral particle preparation, 293T cells were transfected with pCHIV^eYFP ^or with an equimolar mixture of pCHIV^eYFP ^and pCHIV as indicated by standard calcium phosphate precipitation. Tissue culture supernatants were harvested at 40-44 h post transfection, filtered through 0.45 μm filters and particles were concentrated by ultracentrifugation through a 20% (w/w) sucrose cushion. Virus preparations and cell lysates were separated by SDS-PAGE (17.5%, acrylamide:bisacrylamide 200:1) transferred to nitrocellulose and analyzed by ECL immunoblot (Pierce) using polyclonal rabbit antisera raised against purified recombinant His-tagged HIV MA and GFP, respectively. The antiserum raised against GFP displays cross-reactivity with the GFP derivative eYFP. For quantitation of virus by ELISA, tissue culture supernatants or particle preparations, respectively, were diluted with SDS-Sample buffer (final concentration 0.1% SDS) and incubated for 5 min at 95°C to enhance antibody recognition of unprocessed Gag. Samples were subsequently diluted in PBS/0.1% Tween 20 before incubation with capture antibody. The concentration of CA p24 in the samples was determined by ELISA as previously described [32].

Fluorescence measurements of particle preparations were carried out at room temperature in phosphate buffered saline or DMEM/10% FCS as indicated using a TECAN Safire multi-well reader set to excitation and emission wavelengths of 512 nm and 528 nm, or an Aminco-Bowman SLM-2 spectrophotometer for cuvette based measurements, respectively.

### Release assay

293T cells were transfected with pCHIV^eYFP^, pCHIV^eYFP^late(-) or peYFP-C1, respectively, using calcium phosphate or FuGene 6 (Roche) according to standard procedures. At the indicated time points, samples of tissue culture supernatant were removed, briefly centrifuged to remove cell debris (5 min, 3000 g) and subjected to fluorescence measurement. At the end of the indicated incubation period, the remaining medium was removed, cells were briefly washed with phosphate buffered saline (PBS) and incubated in lysis buffer (PBS/0.5% Igepal) at a volume corresponding to the volume of the tissue culture supernatant. Cells were incubated for 10 min at room temperature and cell debris was removed by brief centrifugation (2 min, 1500 g). Fluorescence measurements of supernatants and cell lysates were carried out using an SLM Aminco 2 spectrofluorometer.

For the semi-automated multi-well screening approach, a protocol for batch transfection of cells in suspension was established. 293T cells grown on 10 cm dishes were harvested by trypsinization and adjusted to 3 × 10^5^cells/ml in DMEM without FCS. Per 96-well plate, 960 μl of DMEM without FCS was mixed with 38.4 μl FuGene6 (Roche). Following 5 min incubation at room temperature 19.2 μg of total plasmid DNA were added and the mixture incubated for further 15 min at room temperature. Subsequently, 9.6 ml of the cell suspension were added and 110 μl of the transfection mixture was seeded per well onto 96-well plates (Corning COSTAR #3603) or 25 μl per well onto 384-well plates (BD FALCON 353962, BD Biosciences) using an automated reagent dispenser (MultiDrop^® ^Combi). Plates were incubated at 37°C. At 36 h post transfection, plates were centrifuged (1500 rpm, 8 min) and 87.5 μl of the supernatant was transferred to new 96-well plates (Corning COSTAR #3916) or 20 μl to 384-well plates (BD FALCON 353962) using the compact automated liquid handler Hydra DT (Matrix, Thermo Fisher Scientific). The residual supernatant was removed and 100 μl (96-well plates) or 25 μl (384-well plates) 0.1% Igepal CA630 (Sigma-Aldrich) in PBS were added to each well, followed by one freeze-thaw cycle to promote cell lysis. Fluorescence intensities of supernatants and cell lysates were determined using a multi-well fluorescence reader (Tecan SAFIRE).

Screening of an siRNA library required several modifications of this procedure. The plates used for seeding the cells were pre-coated with an siRNA transfection mixture using a previously described reverse transfection protocol [29,30] and liquid handling platform [33]. Briefly, a mixture of the respective siRNA, the transfection reagent Lipofectamine 2000 (Invitrogen), fibronectin (Sigma-Aldrich), sucrose and gelatine were transferred automatically to 96-well plates (Corning COSTAR #3603). Silencer^® ^Select Negative Control #1 siRNA (Ambion Applied Biosystems) was used as a non-silencing control. For the knock-down of Tsg101 we used siRNA with the sequence 5'CCUCCAGUCUUCUCUCGUC3' selected according to Garrus *et al*. [31]. 293T cells were seeded on top of the dried transfection mixture (7500 cells/well in 100 μL D-MEM 10% FCS) using the reagent dispenser MultiDrop^® ^Combi. Following a 30 hour incubation period at 37°C (18 h for Tsg101, respectively) for target gene knockdown, medium was removed and cells were transfected with an equimolar mixture of plasmids pCHIV and pCHIV^eYFP ^using FuGene6 (Roche) according to the manufacturer's instructions. Per well, 100 ng plasmid DNA in 100 μl medium were added. After an additional 42 h at 37°C (36 h for Tsg101) tissue culture supernatants and cell lysates were harvested and their RFI values were quantitated as described above.

## Abbreviations

HIV: human immunodeficiency virus; ESCRT: endosomal complex required for transport; eYFP: enhanced yellow fluorescent protein; MA: matrix protein; CA: capsid protein; ELISA: enzyme linked immunosorbent assay; RFI: relative fluorescence intensity; PBS: phosphate buffered saline; siRNA: short interfering RNA.

## Authors' contributions

JH adapted the protocol to a semi-automated format, established the protocol for the siRNA screening approach and participated in the design of experiments. MA performed the experiments for the initial characterization and validation of the assay procedure. AMH contributed to adaptation and validation of the semi-automated procedure. BM conceived and coordinated the study, participated in its design, performed initial experiments and drafted the manuscript. All authors have read and approved the final manuscript.

## Supplementary Material

Additional file 1 **Figure S1: Detection of purified fluorescently labelled proteins against medium background**. Fluorescent proteins eGFP (open circles), eYFP (filled circles), eCFP (open triangles) and mRFP1 (open squares), respectively, were expressed as recombinant His-tagged proteins in *Escherichia coli*. Proteins were purified by Ni-chelate affinity chromatography using 1 ml HisTrap columns (GE Healthcare) according to the manufacturer's instructions and dialyzed against PBS. Protein Party was verified by SDS-PAGE and concentrations were calculated from OD280 values determined for GuHCl denatured proteins using extinction coefficients of 21890 (eGFP), 23380 (eYFP), 25900 (eCFP) and 32890 (mRFP), respectively. Serial dilutions were set up in DMEM, 10% FCS, and fluorescence intensity was determined at the appropriate wavelength settings (488/508 nm, 512/528 nm, 433/475 nm and 584/607 nm, respectively) using a TECAN Safire multi-well reader. Background values obtained for medium alone were subtracted. Mean values and standard deviations of triplicate dilutions are shown; lines represent linear regressions. Figure S1 B shows an expansion of the lower part of the graph shown in Figure S1 A. Click here for file

Additional file 2 **Figure S2: Fluorescence spectra of tissue culture supernatants**. Fluorescence emission spectra of tissue culture supernatants harvested at 36 h post transfection from 293T cells transfected with pCHIV^eYFP ^(black solid line), pCHIV^eYFP^late(-) (grey solid line), peYFP-C1 (black dotted line), or from untransfected cells (grey dotted line), respectively, were recorded using an SLM Aminco Spectrofluorometer (excitation wavelength: 512 nm). Click here for file

Additional file 3 **Figure S3: Determination of Z factor from control wells of a pilot screen**. Fluorescence intensities of supernatants from 60 positive control wells (transfected with pCHIV/pCHIV^eYFP^, 0.5% DMSO) and 36 negative control wells (untransfected cells) from 6 replicate 96-well plates were determined. The graph shows mean values and standard deviations (SD) derived from these data. The Z factor [28] was calculated from these data using the formula Z = 1 - (3 × (SD_pos _+ SD_neg_))/(mean_pos _- mean_neg_), yielding a value of 0.71. Click here for file
