# Supplementary material for: A simple fluorescence based assay for quantification of human immunodeficiency virus particle release
Source: BMC Biotechnol. 2010 Apr 20;10:32. doi: 10.1186/1472-6750-10-32 (PMC2873281; doi:10.1186/1472-6750-10-32)
Supplement: Additional file 1 — Figure S1: Detection of purified fluorescently labelled proteins against medium background. Fluorescent proteins eGFP (open circles), eYFP (filled circles), eCFP (open triangles) and mRFP1 (open squares), respectively, were expressed as recombinant His-tagged proteins in Escherichia coli. Proteins were purified by Ni-chelate affinity chromatography using 1 ml HisTrap columns (GE Healthcare) according to the manufacturer's instructions and dialyzed against PBS. Protein Party was verified by SDS-PAGE and concentrations were calculated from OD280 values determined for GuHCl denatured proteins using extinction coefficients of 21890 (eGFP), 23380 (eYFP), 25900 (eCFP) and 32890 (mRFP), respectively. Serial dilutions were set up in DMEM, 10% FCS, and fluorescence intensity was determined at the appropriate wavelength settings (488/508 nm, 512/528 nm, 433/475 nm and 584/607 nm, respectively) using a TECAN Safire multi-well reader. Background values obtained for medium alone were subtracted. Mean values and standard deviations of triplicate dilutions are shown; lines represent linear regressions. Figure S1 B shows an expansion of the lower part of the graph shown in Figure S1 A. [file 1472-6750-10-32-S1.DOC]

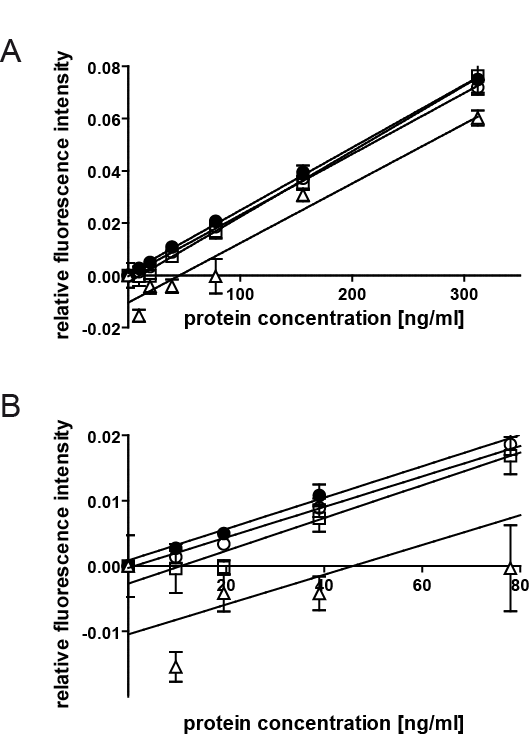


**Figure S1: Detection of purified fluorescently labelled proteins against medium background.** Fluorescent proteins eGFP (open circles), eYFP (filled circles), eCFP (open triangles) and mRFP1 (open squares), respectively, were expressed as recombinant His-tagged proteins in *Escherichia coli*. Proteins were purified by Ni-chelate affinity chromatography using 1 ml HisTrap columns (GE Healthcare) according to the manufacturer’s instructions and dialyzed against PBS. Protein Party was verified by SDS-PAGE and concentrations were calculated from OD280 values determined for GuHCl denatured proteins using extinction coefficients of 21890 (eGFP), 23380 (eYFP), 25900 (eCFP) and 32890 (mRFP), respectively. Serial dilutions were set up in DMEM, 10% FCS, and fluorescence intensity was determined at the appropriate wavelength settings (488/508 nm, 512/528 nm, 433/475 nm and 584/607 nm, respectively) using a TECAN Safire multi-well reader. Background values obtained for medium alone were subtracted. Mean values and standard deviations of triplicate dilutions are shown; lines represent linear regressions. Figure S1 B shows an expansion of the lower part of the graph shown in Figure S1 A.
