# Supplementary material for: A simple fluorescence based assay for quantification of human immunodeficiency virus particle release
Source: BMC Biotechnol. 2010 Apr 20;10:32. doi: 10.1186/1472-6750-10-32 (PMC2873281; doi:10.1186/1472-6750-10-32)
Supplement: Additional file 2 — Figure S2: Fluorescence spectra of tissue culture supernatants. Fluorescence emission spectra of tissue culture supernatants harvested at 36 h post transfection from 293T cells transfected with pCHIVeYFP (black solid line), pCHIVeYFPlate(-) (grey solid line), peYFP-C1 (black dotted line), or from untransfected cells (grey dotted line), respectively, were recorded using an SLM Aminco Spectrofluorometer (excitation wavelength: 512 nm). [file 1472-6750-10-32-S2.DOC]

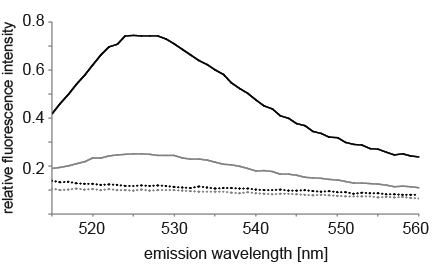


**Figure S2.** **Fluorescence spectra of tissue culture supernatants**. Fluorescence emission spectra of tissue culture supernatants harvested at 36 h post transfection from 293T cells transfected with pCHIV^eYFP^ (black solid line), pCHIV^eYFP^late(-) (grey solid line), peYFP-C1 (black dotted line), or from untransfected cells (grey dotted line), respectively, were recorded using an SLM Aminco Spectrofluorometer (excitation wavelength: 512 nm).
