# Supplementary material for: A simple fluorescence based assay for quantification of human immunodeficiency virus particle release
Source: BMC Biotechnol. 2010 Apr 20;10:32. doi: 10.1186/1472-6750-10-32 (PMC2873281; doi:10.1186/1472-6750-10-32)
Supplement: Additional file 3 — Figure S3: Determination of Z factor from control wells of a pilot screen. Fluorescence intensities of supernatants from 60 positive control wells (transfected with pCHIV/pCHIVeYFP, 0.5% DMSO) and 36 negative control wells (untransfected cells) from 6 replicate 96-well plates were determined. The graph shows mean values and standard deviations (SD) derived from these data. The Z factor [28] was calculated from these data using the formula Z = 1 - (3 × (SDpos + SDneg))/(meanpos - meanneg), yielding a value of 0.71. [file 1472-6750-10-32-S3.DOC]

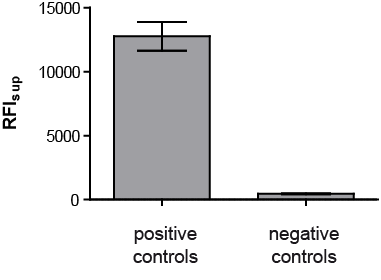


**Figure S3: Determination of Z factor from control wells of a pilot screen.** Fluorescence intensities of supernatants from 60 positive control wells (transfected with pCHIV/pCHIV^eYFP^, 0.5% DMSO) and 36 negative control wells (untransfected cells) from 6 replicate 96-well plates were determined. The graph shows mean values and standard deviations (SD) derived from these data. The Z factor [28] was calculated from these data using the formula Z = 1 – (3 x (SD_pos_ + SD_neg_)) / (mean_pos_ – mean_neg_), yielding a value of 0.71.
